# Monocyte to high-density lipoprotein cholesterol ratio as a marker of the presence and progression of diabetic kidney disease

**DOI:** 10.1080/0886022X.2024.2438846

**Published:** 2025-01-13

**Authors:** Wentao Yang, Yuanlong Zhong, Pengying Zhou, Donghui Lu

**Affiliations:** a Department of Endocrinology, Peking University Shenzhen Hospital, Shenzhen, Guangdong, China; b Department of Nephrology, Shenzhen Luohu People’s Hospital, The Third Affiliated Hospital of Shenzhen University, Shenzhen, Guangdong, China; c Health Management Center, Shenzhen Hospital of Southern Medical University, Shenzhen, Guangdong, China

**Keywords:** Diabetic kidney disease, diabetic nephropathy, monocyte to high-density lipoprotein cholesterol ratio, inflammation, diabetic complications

## Abstract

**Background:**

Monocyte to high-density lipoprotein cholesterol ratio (MHR) is considered a novel marker of inflammation. However, whether MHR can predict the risk of diabetic kidney disease (DKD) remains uncertain. Our research aimed to investigate the relationship between MHR and DKD.

**Methods:**

This was a cross-sectional retrospective study of 159 participants with type 2 diabetes mellitus. MHR, urinary albumin-to-creatinine ratio (UACR) and other indexes were measured. According to UACR, participants were divided into normal group (UACR < 30 mg/g), moderately increased group (UACR 30-300 mg/g) and severely increased group (UACR > 300 mg/g). The association between MHR and DKD was analyzed.

**Results:**

MHR was significantly elevated in severely increased albuminuria group (*p =* 0.029). The prevalence of DKD increased in parallel with the elevation in MHR (*p* = 0.009). MHR was positively related with DKD in univariate logistic regression analysis (ORs = 11.27, 95%CI 1.26-101.24, *p* = 0.031). Multivariable logistic regression analysis showed MHR significantly correlated with DKD (ORs = 6.20, 95%CI 1.49-25.84, *p* = 0.012). Each quartile elevation in MHR was associated with an increased risk of DKD (ORs = 1.90, 95%CI 1.19-3.01, *p* = 0.007). In subgroup analyses MHR was a risk factor for DKD, particularly in patients with HbA1c <8.0%.

**Conclusions:**

Our findings suggest that MHR can be used as a marker for the presence and progression of DKD.

## 1. Introduction

In recent years, the incidence of diabetes mellitus (DM) has substantially increased throughout the world, making it a notable health concern. In 2019, approximately 463 million people suffered from diabetes mellitus, and by 2045, the number of diabetic mellitus cases is expected to reach 700 million, according to the International Diabetes Federation [1]. DM not only results in hyperglycemia, but also leads to plenty of chronic complications, among which diabetic kidney disease (DKD) is one of the most common. DKD affects 20%-40% of patients with DM and accounts for nearly half of patients with end-stage renal disease (ESRD) in developed countries [2]. It usually presents with persistently increased albuminuria with or without renal failure, and eventually progresses to ESRD. The pathogenesis of DKD is comprehensively attributed to elevated formation of glycation end products, oxidative stress and inflammation, fibrosis, elaboration of growth factors, altered glomerular hemodynamics, and hormonal changes [3]. Among these factors, inflammation plays a crucial role.

Monocytes, inflammatory cells that are part of the innate immune response system, are generated from bone marrow and circulate to tissues, where they differentiate into macrophages. In addition to protecting against microbial assault, they play an important role in immune activation. In atherosclerosis, mononuclear phagocytes help take up oxidated low-density lipoprotein into the focal intima *via* unregulated macrophage scavenger receptors, and then transform into inflammatory lipid-laden macrophages (foam cells) that subsequently become atherosclerotic plaques. The mononuclear phagocyte system is also activated in DM. M1 macrophages differentiated from monocytes exhibit the prominent feature of being pro-inflammatory. The accumulation of M1 macrophages has been demonstrated to accelerate endothelial senescence in the diabetic mouse kidney [4]. In addition, Barutta et al. reported that monocyte and M1 macrophage infiltration occur in DKD, which contributes to glomerulosclerosis [5].

The lipid particle high-density lipoprotein cholesterol (HDL-C) is thought to prevent atherosclerosis by protecting against cholesterol oxidation. In contrast to macrophages, which transport cholesterol into atherosclerotic plaques, HDL-C removes cholesterol from plaques, and is therefore considered to be anti-inflammatory. Moreover, HDL-C takes part in the maintenance of endothelial function *via* stimulating endothelial cell proliferation and migration, inhibiting the expression of adhesion molecules, and blunting the expression of monocyte chemoattractant protein-1 [6]. HDL-C may also suppress monocytes, thereby reducing atherosclerosis [7].

As described above, monocytes promote inflammation, whereas HDL-C possesses anti-inflammatory properties. On the basis of existing studies, there is considerable interest in a novel combined inflammatory marker named monocyte to high-density lipoprotein cholesterol ratio (MHR). Increased MHR, which indicates increased monocyte count and reduced HDL levels, reflects higher potential for oxidative stress and inflammation. Previously, several studies have shown that MHR is correlated with atherosclerotic disease [8–11]. Moreover, MHR was also found to be associated with chronic complications of diabetes mellitus such as diabetic retinopathy (DR) [12,13].

Despite these results, the relationship between MHR and DKD was not fully understood. This study aimed to examine the correlation between MHR and DKD, in order to determine whether MHR—a novel noninvasive inflammatory marker—can help recognize patients with DM who are prone to DKD.

## 2. Materials and methods

### 2.1. Subjects

A cross-sectional study was conducted from October 2018 to September 2019.

**Figure 1.**
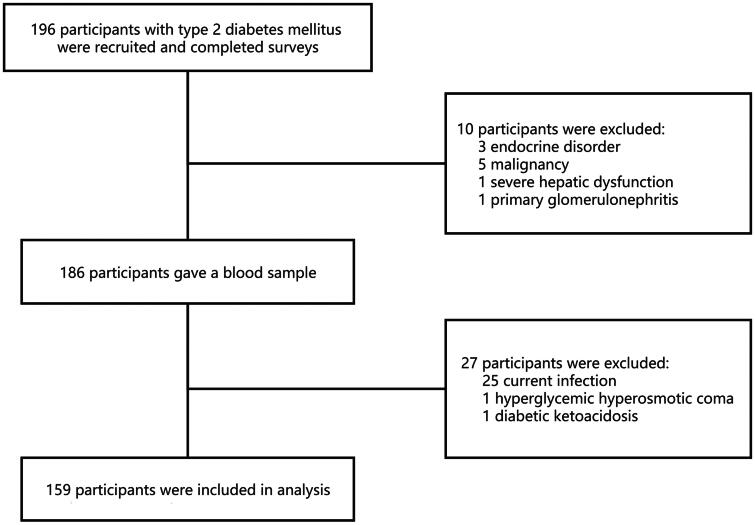
Flowchart of participant inclusion.

We recruited 196 participants with type 2 diabetes mellitus (T2DM) hospitalized in Peking University Shenzhen Hospital, China. A total of 159 participants were retrospectively analyzed. Subjects with other types of diabetes mellitus, diabetic ketoacidosis, hyperglycemic hyperosmotic coma, mental health disorders, current infection, other known causes of kidney damage, severe hepatic dysfunction, congestive heart failure, endocrine disorder, autoimmune disease, or malignancy were excluded. Peking University Shenzhen Hospital’s ethics committee approved the study. All participants gave consent prior to participating in the study. As illustrated in Figure 1, the flow diagram shows the participant selection process.

### 2.2. Physical and laboratory measurements

Body mass index (BMI) was calculated by height and weight. According to the metric system, the BMI formula was defined as weight in kilograms divided by the square of height in meters. After more than 8 h of fasting, venous blood samples were drawn between 6 am and 8 am. Complete blood count was performed using a SYSMEX XN90000-A1 hematology analyzer, whereas creatinine (Cr), blood urea nitrogen (BUN), alanine aminotransferase (ALT), uric acid (UA), glycosylated hemoglobin A1c (HbA1c), fasting glucose (FBG), low-density lipoprotein cholesterol (LDL-C), HDL-C, triglyceride, total cholesterol, and urinary albumin-to-creatinine ratio (UACR) were assessed using a Beckman Coulter AU5831 automatic biochemical analyzer.

### 2.3. Questionnaires

Well-established questionnaires were used to collect information about disease duration, past medical history, tobacco and alcohol intake, current use of angiotensin-converting enzyme inhibitor (ACEI)/angiotensin receptor antagonist (ARB), lipid-lowering therapy, and glucose-lowering medication by well-trained staff through face-to-face interviews.

### 2.4. Definition of monocyte to high-density lipoprotein cholesterol ratio and diabetic kidney disease

The MHR was calculated by dividing the monocyte count (10^3^/L) by the HDL cholesterol level (mg/dL). DKD was defined as persistently increased UACR (>30 mg/g), persistently reduced estimated glomerular filtration rate (eGFR <60 mL/min/1.73 m^2^), or both for greater than three months without other primary causes of kidney injury in patients with DM [14]. There are three categories of albuminuria based on UACR: normal (0-30 mg/g), moderately increased (30-300 mg/g), severely increased (>300 mg/g). On the basis of these established categories, the 159 patients were divided into three groups, including 43 without elevated UACR (A1 group), 73 with moderately increased UACR (A2 group), and 43 with severely increased UACR level (A3 group).

### 2.5. Statistical analysis

The Shapiro-Wilk test as well as histogram and quantile-quantile plot were conducted to verify the normality of variable distribution. Normally distributed continuous variables are expressed as mean ± SD, while values with non-normal distribution are expressed as median with inter-quartile ranges. Categorical variables are presented as numbers (proportions). Comparisons among continuous variables were analyzed by one-way ANOVA or Kruskal-Wallis H test, whereas differences between categorical variables were analyzed by χ^2^ test or Fisher exact test. Post hoc comparisons were performed using Bonferroni correction. DKD and variables were then analyzed using univariate logistic regressions.

The relationship between DKD and each quartile increase in MHR was progressively assessed by multivariable logistic regression. Model 1 was adjusted for age and sex. On the basis of model 1, more confounding variables (duration, HbA1c, BMI, Cr, BUN, UA, hypertension history) were added for model 2. Furthermore, we adjusted model 3 for drugs that affected lipid profiles and drugs with demonstrated benefits for chronic kidney disease, including ACEI/ARB, glucagon-like peptide 1 receptor agonist (GLP-1 RA), and sodium-glucose cotransporter-2 inhibitor (SGLT2i). The association of each quartile elevated MHR with DKD was explored in subgroup analyses and stratified by age (<65 years, ≥65 years), sex, diabetes duration (0-5 years, 6-10 years, >10 years), BMI (<25 kg/m^2^, ≥25 kg/m^2^), hypertension (yes, no), HbA1c (<8.0%, ≥8.0%), Cr (<73 μmol/L, ≥73 μmol/L), BUN (<6.26 mmol/L, ≥6.26 mmol/L), and UA (<420 μmol/L, ≥420 μmol/L). The interaction analyses were used to test the statistically significant differences among different stratifications in subgroups. Missing data were imputed by conducting multiple imputation approaches. Statistical significance was defined as a two-tailed *P* value <0.05 in statistical tests. The statistical analyses were performed using IBM SPSS Statistics 24.0.

## 3. Results

### 3.1. Baseline characteristics of included participants by albuminuria status

**Figure 2.**
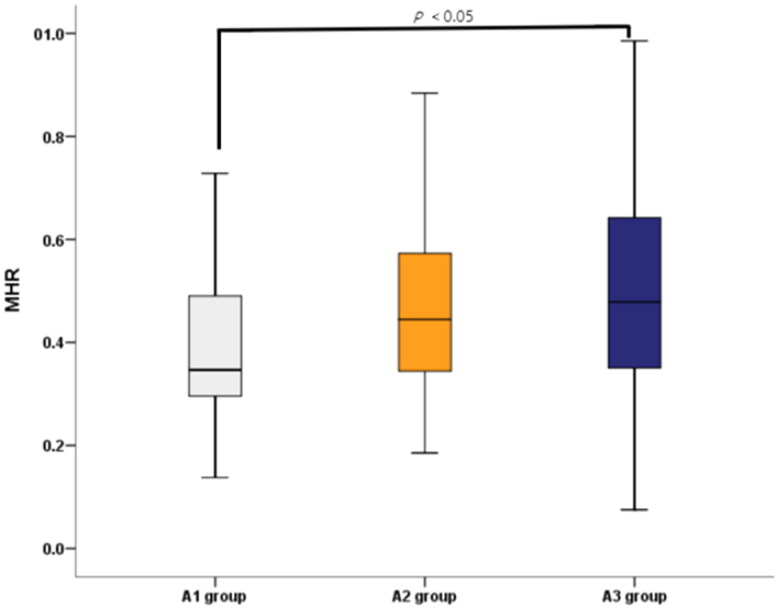
MHR Levels in different groups (A1 group: the normal albuminuria group; A2 group: moderately increased albuminuria group; A3 group: severely increased albuminuria group). MHR: monocyte to high-density lipoprotein cholesterol ratio.

There were totally 159 participants included in our study, with an average age of 58.94 ± 10.47. The general features of participants and their different albuminuria statuses are shown in Table 1. All three groups exhibited similar baseline characteristics. There were no statistical differences among three groups in terms of sex, age, BMI, tobacco use, alcohol abuse, coronary atherosclerotic heart disease (CAD) history, dyslipidemia history, metformin use, GLP-1 RA, SGLT2i or lipid-lowering therapy. As for the laboratory test results, monocyte, HbA1c, FBG, ALT, triglyceride, total cholesterol, HDL-C and LDL-C were also similar across all of the groups. In comparison with the A1 group, MHR was markedly higher in the A3 group (*p =* 0.029, Figure 2), as well as white blood cells. Prevelance of hypertension, ACEI/ARB use, Cr, and UA were increased in the A3 group compared with other groups. Longer diabetes duration, higher frequency of insulin use and higher BUN were observed in both the A2 and A3 groups, when compared with the A1 group (all *p <* 0.05).

**Table 1.** General features of participants by albuminuria status.

Variables	A1 group	A2 group	A3 group	*P*-value
	(*n* = 43)	(*n* = 73)	(*n* = 43)	
Clinical parameters				
** **Male (n (%))	30 (69.8)	44 (60.3)	27 (60.5)	0.550
** **Age (years)	58.69 ± 11.56	58.59 ± 9.46	59.61 ± 11.32	0.946
** **Diabetes duration (years)	8.00 (3.00-11.00)	11.00 (8..00-16.50)*	13.00 (10.00-20.00)*	0.001
** **BMI (kg/m^2^)	24.02 ± 2.47	24.52 ± 3.05	24.16 ± 3.43	0.713
** **Hypertension history (n(%))	22 (51.2)	44 (60.3)	38 (88.4)*^#^	0.001
** **CAD history (n(%))	6 (14.0)	14 (19.2)	12 (27.9)	0.262
** **Dyslipidemia history (n(%))	32 (74.4)	49 (67.1)	34 (79.1)	0.357
** **Smoking (n(%))	8 (18.6)	23 (31.5)	14 (32.6)	0.253
** **Alcohol (n(%))	6 (14.0)	10 (13.7)	3 (7.0)	0.500
Medications				
** **Metformin (n(%))	25 (58.1)	36 (49.3)	15 (34.9)	0.091
** **GLP-1 RA (n(%))	0 (0)	0 (0)	2 (4.7)	0.144
** **SGLT2i (n(%))	0 (0)	3 (4.1)	2 (4.7)	0.524
** **Insulin (n(%))	18 (41.9)	52 (71.2)*	31 (72.1)*	0.003
** **ACEI/ARB (n(%))	6 (14.0)	17 (23.3)	24 (55.8)*^#^	<0.001
** **Lipid-lowering therapy (n(%))	11 (25.6)	20 (27.4)	16 (32.7)	0.428
Laboratory tests				
** **White blood cells (10^9^/L)	6.06 (5.31-7.45)	6.71 (5.67-7.65)	7.27 (5.96-8.09)*	0.042
** **Monocyte (10^9^/L)	0.45 ± 0.15	0.47 ± 0.13	0.47 ± 0.17	0.761
** **HbA1c (%)	8.00 (7.20-9.70)	9.35 (7.05-10.68)	8.4 (7.05-9.70)	0.338
** **Fasting glucose (mmol/L)	7.59 (6.56-8.66)	8.57 (6.93-11.08)	8.36 (6.08-10.41)	0.151
** **Cr (μmol/L)	68.00 (57.00-77.00)	70.00 (56.50-97.50)	121.00 (81.50-97.50)*^#^	<0.001
** **BUN (mmol/L)	4.97 (4.29-6.34)	6.07 (5.22-7.41)*	8.73 (6.30-10.61)*^#^	<0.001
** **UA (μmol/L)	321.00 (281.00-403.00)	366.00 (303.50-428.50)	427.00 (363.00-486.00)*^#^	<0.001
** **ALT (U/L)	18.00 (13.75-27.25)	17.50 (14.00-25.75)	14.00 (12.00-20.50)	0.111
** **Triglyceride (mmol/L)	1.43 (0.98-2.05)	1.53 (1.13-2.62)	2.04 (1.40-2.80)	0.058
** **Total cholesterol (mmol/L)	4.51 (4.01-5.22)	4.74 (3.72-5.92)	4.80 (3.63-5.88)	0.916
** **HDL-C (mmol/L)	1.20 ± 0.34	1.05 ± 0.23	1.05 ± 0.60	0.109
** **LDL-C (mmol/L)	3.07 ± 0.81	3.21 ± 1.08	3.37 ± 1.10	0.410
** **MHR (10^9^/mmol)	0.34 (0.29-0.48)	0.44 (0.35-0.57)	0.46 (0.34-0.64)*	0.029

* Compared with A1 group, *p* < 0.05.

^#^ Compared with A2 group, *p* < 0.05.

BMI: body mass index; CAD history: coronary atherosclerotic heart disease history; GLP-1 RA: glucagon-like peptide 1 receptor agonist; SGLT2i: sodium-glucose cotransporter-2 inhibitor; ACEI: angiotensin-converting enzyme inhibitor; ARB: angiotensin receptor antagonist; HbA1c: glycosylated hemoglobin A1c; Cr: creatinine; BUN: blood urea nitrogen; UA: uric acid; ALT: alanine aminotransferase; HDL-C: High-density lipoprotein cholesterol; LDL-C: low-density lipoprotein cholesterol; MHR: monocyte to high-density lipoprotein cholesterol ratio.

### 3.2. Association between MHR and DKD

**Figure 3.**
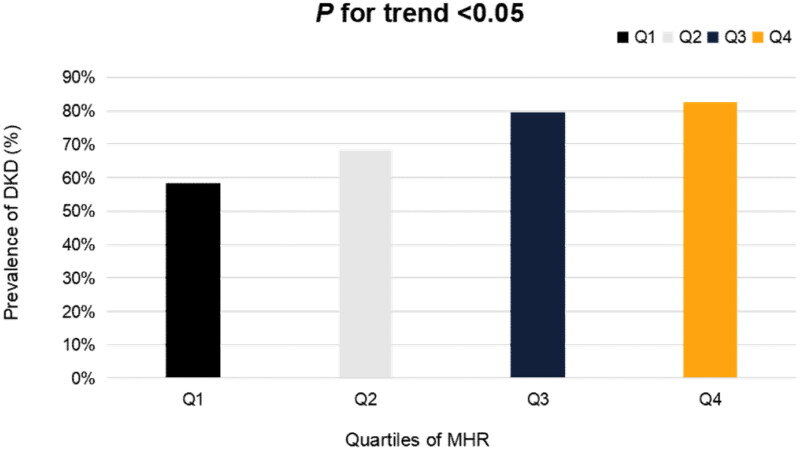
DKD Prevalence in different MHR quartiles (Q1: 0.08-0.32; Q2: 0.33-0.42;Q3: 0.43-0.57; Q4: 0.58-0.99).

The prevalence of DKD was 58.5%, 68.4%, 79.5%, and 82.5%, respectively, in the different MHR quartiles, increasing in parallel with the elevation in MHR (*p* = 0.009) (Figure 3). MHR correlated positively with DKD in univariate logistic regression analysis (Table 2, *p* = 0.031). In addition, diabetes duration, Cr, BUN, UA, hypertension history, and ACEI/ARB therapy were positively associated with DKD. The further relationship between DKD and MHR was then explored by multivariable logistic regression (Table 3). In model 1, significantly increased DKD ORs were observed in MHR quartile 3 (OR = 2.15, 95%CI: 1.28–10.88, *p* = 0.016) and MHR quartile 4 (OR = 4.02, 95%CI: 1.37–11.78, *p* = 0.011) versus MHR quartile 1 (reference), independent of age and sex. In model 2, after adjustment for not only age and sex, but also diabetes duration, hypertension, HbA1c, BMI, Cr, BUN, and UA, risk of DKD with OR of MHR quartile 3 was 4.53 (95%CI: 1.09-18.82, *p* = 0.038) and the one of MHR quartile 4 was 6.20 (95%CI: 1.49-25.84, *p* = 0.012), respectively. Moreover, ORs in model 3 were consistent with model 2, even after further adjusting for medications, including lipid-lowering therapy, ACEI/ARB, SGLT2i, and GLP-1 RA. Each quartile elevation in MHR was correlated with a raised risk of DKD in model 1 (OR = 1.53, 95%CI 1.1–2.12, *p* = 0.012) as well as models 2 and 3 (ORs = 1.90, 95%CI 1.19-3.01, *p* = 0.007). In multivariable logistic regression models above, there was no multicollinearity (tolerance *>* 0.1, variance inflation factor < 10). No obvious outlier, high leverage points and strong influence points were observed (studentized residual <2SD). Hosmer and Lemeshow Goodness-of-fit Tests were conducted and the results showed *p* = 0.562 in model 1, *p* = 0.325 in model 2-3, which confirmed the validity of the models.

**Table 2.** Univariate logistic regression analysis for the presence of diabetic kidney disease.

Variables	OR (95%CI)	*P*-value
MHR (10^9^/mmol)	11.27 (1.26-101.24)	0.031
Age (years)	1.01 (0.97-1.04)	0.738
Male	0.66 (0.31-1.40)	0.276
Diabetes duration (years)	1.13 (1.05-1.20)	0.001
HbA1c (%)	1.1 (0.9-1.4)	0.206
BMI (kg/m^2^)	1.05 (0.93-1.18)	0.463
Cr (μmol/L)	1.03 (1.01-1.05)	<0.001
BUN (mmol/L)	1.65 (1.29-2.10)	<0.001
UA (μmol/L)	1.01 (1.00-1.01)	0.001
Hypertension	2.30 (1.12-4.73)	0.023
ACEI/ARB	3.37 (1.31-8.66)	0.012
Lipid-lowering therapy	1.31 (0.59-2.88)	0.504

MHR: monocyte to high-density lipoprotein cholesterol ratio; HbA1c: glycosylated hemoglobin A1c; BMI: body mass index; Cr: creatinine; BUN: blood urea nitrogen; UA: uric acid; ACEI: angiotensin-converting enzyme inhibitor; ARB: angiotensin receptor antagonist.

**Table 3.** Multivariable logistic regression analysis for the presence of diabetic kidney disease.

Variable	Model 1†		Model 2‡		Model 3§	
	OR (95%CI)	*P*	OR (95%CI)	*P*	OR (95%CI)	*P*
MHR						
Q1	Reference		Reference		Reference	
Q2	1.60 (0.63-4.11)	0.325	1.76 (0.52-5.94)	0.366	1.76 (0.52-5.94)	0.366
Q3	3.73 (1.28-10.88)	0.016	4.53 (1.09-18.82)	0.038	4.53 (1.09-18.82)	0.038
Q4	4.02 (1.37-11.78)	0.011	6.20 (1.49-25.84)	0.012	6.20 (1.49-25.84)	0.012
One quartile increase	1.53 (1.1-2.12)	0.012	1.90 (1.19-3.01)	0.007	1.90 (1.19-3.01)	0.007

† Model 1: adjusted for age and sex.

‡ Model 2: adjusted for age, sex, diabetes duration, hypertension, HbA1c, BMI, Cr, BUN, and UA.

§ Model 3: adjusted for age, sex, diabetes duration, hypertension, HbA1c, BMI, Cr, BUN, UA, ACEI/ARB, SGLT2i, GLP-1 RA, and lipid-lowering therapy.

### 3.3. Subgroup analysis of the association between MHR and DKD

**Figure 4.**
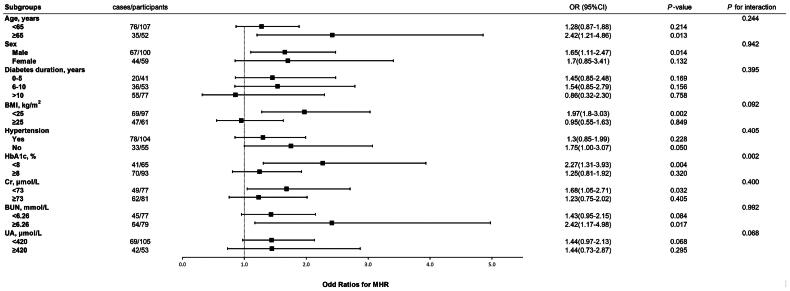
Test for interaction test between each quartile elevation of MHR and DKD in subgroups. MI: body mass index; HbA1c: glycosylated hemoglobin A1c; Cr: creatinine; BUN: blood urea nitrogen; UA: uric acid.

The risk of DKD with OR of each quartile elevation of MHR in the different subgroups, as well as the interactions among the subgroups, is shown in Figure 4. The interactions with HbA1c suggested that MHR was a risk factor for DKD particularly when HbA1c <8.0% (OR = 2.27, 95%CI: 1.31–3.93 *p* = 0.004).

## 4. Discussion

The relationship between MHR and diabetic kidney disease is not yet fully understood. In our study, we found that MHR was positively correlated with DKD, and that MHR was a risk factor for DKD independent of various conventional confounding variables.

As mentioned earlier, inflammation plays an essential role in DKD progression. MHR, an inflammatory indicator, is a novel but easily assessed and reliable biomarker. Previous studies have yielded conflicting results as to the relationship between MHR and chronic complications of diabetes mellitus, especially regarding microvascular complications. Tang et al. suggested that MHR could be used to predict DR in patients with T2DM [13]. In addition, Yalinbas Yeter et al. reported that MHR can be used to predict diabetic macular edema with high sensitivity and specificity [15]. However, Gökçay Canpolat et al. found no association between MHR and the presence of diabetic peripheral neuropathy [12]. Given the growing interest in DKD, a few studies of MHR and DKD have been performed. Onalan et al. and Efe et al. both found that patients with DKD had increased MHR, but they were unable to demonstrate a significant correlation between albuminuria and MHR, or provide the analyses of associations with DKD by multivariable regression [16,17]. Karatas A et al. showed that patients with DKD had elevated MHR values, and there was a positive relationship between albuminuria and MHR after adjusting for several confounding variables (FBG, Cr, triglyceride, HDL-C, WBC, hemoglobin, monocyte, CRP, and UA; but not HbA1c or duration of disease) [18]. In line with our hypothesis, our findings demonstrate that there was a statistically significant increase in MHR in diabetic patients with macroalbuminuria compared with those without macroalbuminuria. MHR was correlated positively with DKD and found to be an independent risk factor for DKD after adjustment for multiple confounders (age, sex, diabetes duration, hypertension, HbA1c, BMI, Cr, BUN, UA). However, none of those previous studies concerning diabetic kidney disease had taken the impacts of medications into account. We are the first to notice that MHR remains a risk factor for DKD even in patients taking medications with renoprotective effects (ACEI/ARB, SGLT2i, GLP-1 RA) and lipid-lowering medications that could influence HDL-C levels.

Interestingly, further subgroup analyses exhibited that the association between MHR and DKD was especially statistically significant in patients without poor glycemic control (HbA1c <8.0%). The American Diabetes Association Standards of Medical Care in Diabetes-2022 state that it is reasonable to set less stringent HbA1c goals (such as 8.0%) for patients whose life expectancy are limited or if the disadvantages of tight glycemic control outweigh the advantages [19]. Therefore, HbA1C ≧8% is recognized as poor glycemic control in the overwhelming majority of patients with DM. There is substantial evidence that poor glycemic control leads to a higher prevalence of chronic complications of diabetes [14]. Several large, well-known trials have focused on the effects of improved glycemic control. Regarding T2DM, United Kingdom Prospective Diabetes Study (UKPDS) demonstrated reductions in all microvascular events by 25% in the intensive glycemic control arm. The T2DM trial Action in Diabetes and Vascular Disease: Preterax and Diamicron Modified Release Controlled Evaluation (ADVANCE) revealed a relative risk reduction of 14% for microvascular events, a relative risk reduction of 21% for diabetic kidney disease, and a relative risk reduction of 65% for ESRD linked to intensive glycemic control. A meta-anlaysis including four known large-scale randomized controlled trials (ACCORD, ADVANCE, UKPDS, and VADT) showed that with the relative risk of renal events was reduced by 20% with more intense glucose management during follow-up period [20]. Similarly, the Epidemiology of Diabetes Interventions and Complications (EDIC) Study and the Diabetes Control and Complications Trial (DCCT) both observed that intensive glycemic control resulted in a reduction of albuminuria in type 1 diabetes mellitus as well. Since the development of DKD is not only caused by inflammation, but also results from other factors, such as advanced glycation end-products (AGEs) [3]. Glycation of proteins and other molecular structures occurs as a result of hyperglycemia, culminating in irreversibly glycated forms known as AGEs. The extent of AGE production and receptor for AGEs (RAGE) expression in glomerular and tubulointerstitial compartments are correlated with the severity of DKD. AGEs and RAGE exert their effects on the development of DKD by deregulating the balance between synthesis and degradation of extracellular matrix components, inducing podocytopathy and mesangiopathy, enhancing angiotensin II activity, inducing renal inflammation and fibrosis [21]. AGEs’s interaction with the AGE-specific receptor on monocytes and macrophages could stimulate the secretion of cytokines and mediate the migration of the monocytes, promoting inflammation [22].

We also found that history of hypertension, ACEI/ARB use, insulin use and elevated Cr, BUN, and UA levels, which are often associated with kidney disease, were more common in the severely increased albuminuria group than in the other two groups. Although there was a tendency for monocyte counts to rise as albuminuria increased, consistent with Onalan E’s findings [16], this association did not reach statistical significance, probably due to small sample size of our study. However, WBC counts were found to be significantly elevated in the severely increased albuminuria group, implying that chronic inflammation contributes to DKD.

Our study had several strengths. MHR is a convenient biomarker that can be easily calculated using data from inexpensive, traditional, and well-established blood tests. Our findings show that elevated MHR values are related to an escalated risk of DKD, suggesting that MHR can be used to indicate the presence and progression of DKD without assessing albuminuria or eGFR in initial screening or common physical exam, thus reducing medical costs. Specifically, the multivariable-adjusted models used in our study not only focused on demographic characteristics, past histories, and laboratory tests, but also took medications into consideration.

Certain limitations should also be considered. First, this was a single-center study. Second, the retrospective cross-sectional study design identified a correlation but could not establish causation. Third, sample size was not large, which may increased the probability of a type I error. Fourth, other inflammatory biomarkers like C-reactive protein, IL-6, procalcitonin were not assessed. Last, because a diagnosis of DKD can be made in clinical practice based on albuminuria or eGFR in the absence of other kidney damage, the patients included in our study were diagnosed with DKD without kidney biopsy [23].

To summarize, MHR, a novel and readily available biomarker, increases in parallel with DKD progression. MHR was considered as an independent risk factor for DKD, particularly in the absence of poor glycemic control. Furthermore, MHR was significantly elevated in diabetic patients with severely increased albuminuria, suggesting aggravation of DKD. This relationship between MHR and DKD provides new insights into the early identification of DKD presence and progression in clinical management.

## Approval of the research protocol

This study was granted ethical approval by Peking University Shenzhen Hospital and was conducted in accordance with the principles outlined in the Declaration of Helsinki.

## Informed consent

The participants provided written informed consent to participate in this study. No potentially identifiable human images or data are presented in this study.
